# Cryogel-supported stem cell factory for customized sustained release of bispecific antibodies for cancer immunotherapy

**DOI:** 10.1038/srep42855

**Published:** 2017-02-16

**Authors:** Roberta Aliperta, Petra B. Welzel, Ralf Bergmann, Uwe Freudenberg, Nicole Berndt, Anja Feldmann, Claudia Arndt, Stefanie Koristka, Marcello Stanzione, Marc Cartellieri, Armin Ehninger, Gerhard Ehninger, Carsten Werner, Jens Pietzsch, Jörg Steinbach, Martin Bornhäuser, Michael P. Bachmann

**Affiliations:** 1 Helmholtz-Zentrum Dresden-Rossendorf (HZDR), Institute of Radiopharmaceutical Cancer Research, Bautzner Landstrasse 400, 01328 Dresden, Germany; 2 Leibniz Institute of Polymer Research Dresden (IPF), Hohe Strasse 6, 01069 Dresden, Germany; 3 Institute of Physiological Chemistry, Technische Universität Dresden, Fetscherstrasse 74, 01307 Dresden, Germany; 4 Cellex Patient Treatment GmbH, Tatzberg 47, 01307 Dresden, Germany; 5 University Cancer Center (UCC), Technische Universität Dresden, Tumorimmunology, Fetscherstrasse 74, 01307 Dresden, Germany; 6 GEMoaB Monoclonals GmbH, Tatzberg 47, 01307 Dresden, Germany; 7 Medical Clinic and Policlinic I, University Hospital ‘Carl Gustav Carus’, Technische Universität Dresden, Fetscherstrasse 74, 01307 Dresden, Germany; 8 DKTK (German consortium for Translational Cancer Research), Dresden, Germany; 9 National Center for Tumor Diseases (NCT), Dresden, ‘Carl Gustav Carus’ TU Dresden, Dresden, Germany; 10 DFG-Center for Regenerative Therapies Dresden, Technische Universität Dresden, Fetscherstrasse 105, 01307 Dresden, Germany; 11 Department of Chemistry and Food Chemistry, School of Science, Technische Universität Dresden, 01069 Dresden, Germany

## Abstract

Combining stem cells with biomaterial scaffolds provides a promising strategy for the development of drug delivery systems. Here we propose an innovative immunotherapeutic organoid by housing human mesenchymal stromal cells (MSCs), gene-modified for the secretion of an anti-CD33-anti-CD3 bispecific antibody (bsAb), in a small biocompatible star-shaped poly(ethylene glycol)-heparin cryogel scaffold as a transplantable and low invasive therapeutic machinery for the treatment of acute myeloid leukemia (AML). The macroporous biohybrid cryogel platform displays effectiveness in supporting proliferation and survival of bsAb-releasing-MSCs overtime *in vitro* and *in vivo*, avoiding cell loss and ensuring a constant release of sustained and detectable levels of bsAb capable of triggering T-cell-mediated anti-tumor responses and a rapid regression of CD33^+^ AML blasts. This therapeutic device results as a promising and safe alternative to the continuous administration of short-lived immunoagents and paves the way for effective bsAb-based therapeutic strategies for future tumor treatments.

Immunotherapy of tumors, i.e. harnessing the immune system for therapeutic benefit in cancer, has gained much prominence in the last years^1^. The main strategies are based on boosting the immune response via a plethora of compounds, such as antibodies, chemokines, vaccines or *ex vivo* stimulated immune effector cells^2^. However, for their wide-spread use several challenges with respect to pharmacokinetics, efficiency and safety still need to be met^3^,^4^. Recent studies have demonstrated that combining cancer immunotherapy with biomaterials may help to address some of these limitations^3^,^5^. A wide variety of scaffolds and hydrogel-based platforms made of synthetic and natural materials, capable to modulate the immune response against tumors, have been described during the last decades^6^. For instance, biomaterials have been employed as devices for controlled delivery of active molecules and cells, or as engineered microenvironments for recruiting and programming immune cells *in situ*^3^.

Here, we report an advanced approach for developing an immunotherapeutic organoid by housing human mesenchymal stromal cells (MSCs), genetically modified for the production of bispecific antibodies (bsAbs) in implantable, mechanically robust, sponge-like glycosaminoglycan (GAG)-based hydrogels (cryogels)^7^,^8^,^9^. The anti-tumor effectiveness of bsAbs is given by their capacity to increase specificity but also to enhance potency of conventional tumor therapy by selectively binding to a specific tumor associated antigen (TAA) on malignant cells and an activating CD3-complex on effector T-cells^10^,^11^,^12^,^13^,^14^. Given their impressive success in pre-clinical and clinical trials^15^,^16^,^17^, we hypothesized that the development of an artificial bsAb-bioreactor, allowing constant *in vivo* secretion of these therapeutic agents, would further enhance the effectiveness of bsAbs-based tumor treatments. In this context, recently introduced macroporous four-arm poly(ethylene glycol) (starPEG)-heparin cryogels^7^,^8^,^9^ (Fig. 1) would potentially provide bsAb-secreting cells with a biomimetic microenvironment allowing for their proper attachment, preventing their escape and enabling effective transport of therapeutic antibodies, nutrients, and metabolites, meanwhile protecting housed cells from mechanical stress^9^. This cryogel-supported cell factory is expected to permit customized and sustained release of bsAbs, overcoming relevant limitations associated with administration of soluble bsAbs or injection of *ex vivo* gene-modified bsAb-secreting cells, such as frequent re-dosing, systemic toxicity, cell loss and high costs^18^,^19^,^20^,^21^,^22^. Moreover, the suggested strategy would ensure that the delivery of bsAbs could be controlled and therefore blocked once the therapeutic effect is fulfilled by removing the cell-laden biomimetic cryogel matrix from its implantation site as needed.

As a proof-of-concept prototype, we report the development of a cryogel-supported stem cell factory suitable for the treatment of acute myeloid leukemia (AML) via constant and long-lasting delivery of a fully humanized anti-CD33-anti-CD3 bsAb, capable of specifically and efficiently redirecting CD3^+^ T lymphocytes towards CD33^+^ AML blasts^14^,^23^.

## Methods

### Ethics statement

Human peripheral blood mononuclear cells (PBMCs) were isolated either from buffy coats supplied by the German Red Cross (Dresden, Germany) or from fresh blood of healthy donors. A written informed consent was obtained from all subjects.

All the methods concerning the use of human samples were carried out in accordance with relevant local guidelines and regulations. This study, including the consent form from human healthy donors, was approved by the local ethics committee of the university hospital of the medical faculty of Carl-Gustav-Carus, Technische Universität Dresden, Germany (EK27022006).

All animal experiments performed in the present study were carried out at the Helmholtz-Zentrum Dresden-Rossendorf according to the guidelines of German Regulations for Animal Welfare. All the methods and protocols pertaining to animal experiments were approved by the Governmental IACUC (‘Landesdirektion Sachsen’) and overseen by the animal ethics committee of the Technische Universität Dresden, Germany (reference numbers 24D-9168.11-4/2007-2 and 24-9168.21-4/2004-1).

### Macroporous starPEG-heparin cryogel scaffolds

The fabrication of starPEG-heparin cryogel scaffolds has been described elsewhere^7^,^8^. Briefly, network formation via chemical crosslinking (EDC/sulfo-NHS chemistry) of 4-arm amino terminated starPEG (molecular mass 10,000 g/mol; JenKem Technology, USA) and heparin (molecular mass 14,000 g/mol; Merck, Germany) was combined with cryogelation technology. The aqueous reaction mixture was pipetted into the cavities of a 96-well plate (350 μl per well) and frozen at −20 °C overnight, before the samples were lyophilized for 24 h^7^,^8^. For the present study a molar ratio of starPEG to heparin of γ = 1.5 and a total precursor concentration of 11.7% (w/w) was used. Some cryogels were fluorescently labeled by mixing heparin with 1% (w/w) of Alexa Fluor^®^ 647-labeled heparin (prepared from Alexa Fluor^®^ 647, Gibco, UK). The resulting dry cryogel cylinders were cut into discs with 1 mm height and punched in discs of 3 mm diameters with a punching tool (Hoffmann GmbH, Qualitätswerkzeuge, München, Germany). The discs (in the following: scaffolds) were washed and swollen in phosphate buffered saline (PBS, pH 7.4) as previously described^7^ to also remove EDC/sulfo-NHS and any unbound starPEG/heparin.

The mechanical and architectural properties of the PBS swollen cryogel scaffolds were reported elsewhere^7^,^8^,^24^,^25^. The morphological features of the dry starPEG-heparin cryogel scaffolds were examined by scanning electron microscopy and the pore size distribution in the swollen state was determined from cross-sectional confocal images of fluorescently labeled cryogels^7^,^9^.

To improve cell adhesion, the starPEG-heparin cryogel scaffolds were biofunctionalized with an RGD (Arg-Gly-Asp) containing peptide sequence (H_2_N-GWGGRGDSP-CONH_2_, molecular mass 886.92 g/mol). Therefore, the PBS swollen scaffolds were first sterilized with ProClin (Supelco, USA, 0.04% in PBS) overnight and, following three washing with PBS, carboxylic acid groups of heparin were activated with EDC/sulfo-NHS solution (50 mM EDC, 25 mM sulfo-NHS in 67 mM phosphate buffer (pH 5)) for 1 h. Subsequently, the scaffolds were washed three times in borate buffer (100 mM, pH 8, 4 °C) to remove unbound EDC/sulfo-NHS and then incubated in 300 μL H_2_N-GWGGRGDSP-CONH_2_-solution respectively, dissolved in borate buffer for 3 h at room temperature, washed in PBS three times, and maintained at 4 °C in sterile PBS until use.

### Cell lines and scaffold seeding

The human bone marrow-derived mesenchymal stromal cell (MSC) line SCP-1^26^ was genetically modified by lentiviral transduction for the ectopic expression of the eGFP cassette and for the stable production of the anti-CD33-anti-CD3 bispecific antibody (bsAb)^14^ or surface expression of 4-1BB ligand as recently described^22^. The wild type (wt), vector control, bsAb-producing or bsAb-producing/4-1BBL-expressing modified MSCs and the human acute myeloid leukemia (AML) cell lines MOLM-13 and MOLM-13-Luc^+^ (see below) were maintained at 37 °C and 5% CO_2_ in RPMI 1640 medium (10% FCS and 100 μg/ml penicillin/streptomycin). To express the enzyme luciferase (*Photinus pyralis*) useful for *in vivo* optical imaging (see below) of MOLM-13 cells, the AML cell line was transduced with the open reading frame encoding the firefly luciferase gene as previously described^14^. The resulting cells were termed as MOLM-13-Luc^+^ cells. All cell lines were purchased from the Leibniz-Institut-DSMZ German collection of microorganisms and cell cultures.

Prior to MSCs seeding, starPEG-heparin cryogel scaffolds (80 μg/ml RGD peptide) were dried out on sterile filter papers to remove excess of PBS from the macropores. Concentrated cell solutions (10 μl) containing different cell numbers (5 × 10^3^, 1 × 10^4^, 5 × 10^4^, 5 × 10^5^) in complete RPMI were evenly added on top of each scaffold, and incubated at 37 °C and 5% CO_2_ for 1 h without additional medium to allow a complete absorption of the cell suspension and to enhance cell adhesion. Samples were subsequently washed with culture medium to remove unattached cells and transferred to a fresh 96-well plate.

### *In vitro* proliferation of cryogel-housed MSCs

The seeding efficiency and the proliferation of gene-modified MSCs seeded into starPEG-heparin cryogel scaffolds were assessed via Alamar blue assay. Briefly, Alamar blue reagent (AbD Serotec, Oxford, UK) was added into the cell culture medium (10%) of either MSCs/cryogel samples or conventional 2D MSCs cultures used as control. Both cell cultures were seeded in triplicates in 96-well plates at comparable cell concentrations. The colorimetric absorbance of the reduced Alamar blue was measured using a Sunrise^TM^ Microplate Reader (Tecan, Maennedorf, Switzerland) at excitation wavelengths of 570 nm and 600 nm after different incubation time points. The percentage of Alamar blue reduction was calculated according to the manufacturer’s instructions. Moreover, the optical density of the Alamar blue reduced by the MSCs/cryogel samples was compared with that of control 2D cultures to determine the initial seeding efficiency in percentage for each MSCs concentration and roughly quantify total number of modified-MSCs proliferating in the scaffold over prolonged culture time.

### Fluorescence microscopy and scaffold sectioning

For fluorescence microscopy analysis the MSCs/cryogel samples were rinsed in PBS and fixed with 3.6% formaldehyde (Sigma-Aldrich, Steinheim, Germany) buffered with 100 mM sodium phosphate at pH 7.4 for 20 min at room temperature. Fixed samples were washed with PBS and incubated first overnight in 30% (w/v) sucrose solution at 4 °C, then in 15% (w/v) sucrose solution and subsequently in 50% (v/v) Tissue Tek “O.C.T.” (Sakura Finetek Europe, Zoeterwoude, The Netherlands), before they were frozen on dry ice in 100% “O.C.T.” compound. Serial sections of 8 μm thickness were cut using a Leica CM 1900 cryostat (Leica Microsystems, Nussloch, Germany) and dried onto glass slides. Scaffold sections were then permeabilized using 0.2% Triton-X-100 (Sigma-Aldrich) in PBS and blocked in 1% BSA/PBS at room temperature for 30 minutes. Actin filaments were stained using Alexa Fluor^®^ 647 Phalloidin (Life technologies, Carlsbad, CA) (1:200) and the sections were mounted in Slowfade^®^ Gold (Life technologies) antifade mounting media with DAPI (Thermo Scientific, Waltham, Massachusetts, USA). Stained samples were visualized with Zeiss Axiophot fluorescence microscope equipped with an AxioCam MRm camera and images were analyzed using AxioVision software (all Zeiss, Oberkochen, Germany).

### Cell viability and TUNEL assay

The viability of gene-modified MSCs housed in cryogels was assessed via TUNEL assay using the *In Situ* Cell Death Detection Kit (Roche Diagnostics, Mannheim, Germany). Different numbers of modified MSCs (1 × 10^4^ and 5 × 10^4^ cells) were cultured on starPEG-heparin cryogels for 10 days. Subsequently three scaffolds for each seeding density were cryo-sectioned as described above and incubated with the TUNEL reaction mixture following the manufacturer’s instruction. The apoptotic index was calculated as the percentage of TUNEL-positive cells on the total number of cells counted in five random fields for each experimental triplicate using a Zeiss Axiophot fluorescence microscope equipped with an AxioCam MRm camera (both Zeiss). TUNEL-positive but DAPI-negative cells were considered as artifacts and excluded from the analysis.

### Flow cytometry analysis

BsAb-releasing MSCs were analyzed for the expression of either CXCR4 or TNFR1 receptors. For extracellular staining, cells were incubated with anti-CXCR4/PE or anti-TNFR1/PE (both Miltenyi Biotec, Bergisch-Gladbach, Germany) monoclonal antibodies (mAbs) at 4 °C for 30 min. For intracellular staining, the extracellular CXCR4 and TNFR1 receptors were first blocked with 10 μg/mL anti-human CXCR4 or anti-human TNFR1 (both BioLegend, Fell, Germany) mAbs respectively at 4 °C for 1 h. Subsequently, samples were fixed with 4% formaldehyde (Sigma-Aldrich), permeabilized with 0.5% Triton-X-100 (Sigma-Aldrich) and stained with anti-CXCR4/PE or anti-TNFR1/PE (Miltenyi Biotec) mAbs as reported above. Samples were analyzed using a MACSQuant Analyzer^®^ and MACSQuantify software (both Miltenyi Biotec).

### Transwell migration assay

Chemotaxis assay was performed in 24-well Corning^®^ Costar^®^ transwell chambers with polycarbonate membranes with pore sizes of 5 μm (Fisher Scientific, Rockville, USA). Equivalent numbers (1 × 10^5^) of gene-modified MSCs were either housed in starPEG-heparin cryogels functionalized with different RGD peptide concentrations (40 μg/ml, 80 μg/ml or 160 μg/ml) or seeded in 2D in the upper chambers in complete RPMI media. The lower compartments were filled either with complete media, or 150 ng/ml SDF-1α (Sigma-Aldrich), or 100 ng/ml TNF-α (PeproTech, Hamburg, Germany) as chemoattractants. Samples were incubated in normoxic incubator for 20 h at 37 °C. Subsequently, the cells from the top side of the membrane insert were removed and migrated cells on the bottom side of the membrane were fixed with 4% formaldehyde (Sigma-Aldrich), stained with 1% crystal violet (Sigma-Aldrich) for 10 min and then visualized using a Zeiss Axiovert 40 CFL microscope equipped with an AxioCam HR camera (both Zeiss).

### Colony formation assay

Gene-modified MSCs were cultured at different seeding numbers (5 × 10^3^, 1 × 10^4^, 5 × 10^4^) in triplicate in 96-well plates either in 2- or 3-dimensions for an overall time of 10 days. At fixed time points culture media were sucked off, cryogel samples were removed and the wells housing respective samples were washed with PBS, fixed with 4% formaldehyde (Sigma-Aldrich) at room temperature for 10 min and then stained with 0.1% crystal violet (Sigma-Aldrich) for 20 min. Samples were subsequently thoroughly washed with deionized water and dried out on filter paper. Blue dye was dissolved in 100 μl methanol and emission spectra were measured at 590 nm using a Sunrise^TM^ Microplate Reader (Tecan).

### *In vitro* release of CD33-CD3 bsAb from cryogel-housed MSCs

Culture supernatants (200 μl) from bsAb-producing MSCs seeded at different densities (5 × 10^3^, 1 × 10^4^, 5 × 10^4^/scaffold) on starPEG-heparin cryogel scaffolds were harvested at reported time points, spun down (360 × g, 5 min) and frozen until analysis. The same volume of fresh cell culture medium was replaced. Subsequently, collected supernatants were analyzed for bsAb release by enzyme-linked immunosorbent assay (ELISA) as recently described^22^. Standards and samples were run in duplicates. The optical density at 450 nm was used to evaluate the concentration of the MSC-released bsAb and the cumulative release was calculated as the sum of bsAb released at each time point.

### Isolation of human T-lymphocytes from healthy donors

Human CD3^+^ T-cells were freshly isolated from human peripheral blood mononuclear cells (PBMCs) derived from healthy volunteers after their informed consent by negative selection using the pan T-cell isolation kit (Miltenyi Biotec) and cultured in complete RPMI 1640 medium containing 50 U/ml IL-2 (ImmunoTools, Friesoythe, Germany)^11^.

### T-cell activation assay

Freshly isolated human pan T-cells co-incubated with 1 × 10^4^ CD33^+^ MOLM-13 cells at an effector-to-target (E:T) cell ratio of 1:1 in the presence or absence of 1 × 10^4^ bsAb-releasing MSCs seeded either in 2- or 3D were analyzed for the surface expression of specific T cell activation markers CD25 and CD69^22^. After 48 h of co-culture, the cells of one triplicate were pooled and stained with a mixture of anti-CD3/VioBlue, anti-CD69/FITC (all purchased from Miltenyi Biotec) and anti-CD25/PE (BD Biosciences, Heidelberg, Germany) mAbs.

### *In vitro* tumor cell killing via bsAb-redirected T-cells

The CD33^+^ AML cell line MOLM-13 was co-incubated in the presence or absence of 1 × 10^4^ CD3^+^ T-cells at an E:T cell ratio of 1:1 together with 1 × 10^4^ bsAb-releasing or bsAb-releasing/4-1BBL expressing MSCs pre-seeded for 48 h on cryogel scaffolds or as conventional 2D culture as control in 96-well plates. Specific tumor cell lysis derived from the cross-linkage between tumor target cells and T-cells via the bsAb released by the modified MSCs was examined by standard ^51^Cr-release cytotoxicity assays as described elsewhere^11^.

### Animal experiments

In the present study a total of 39 NMRI^nu/nu^ mice were used. Animals were kept under standardized conditions with autoclaved food, water, and bedding.

A total of 22 NMRI^nu/nu^ mice were subcutaneously (s.c.) implanted into the left leg with Cryogels housing bsAb-releasing MSCSs (5 × 10^5^), whereas 2 × 10^6^ CD33^+^ MOLM-13 cells were s.c. injected into their right legs. Following tumor inoculation, tumor size was measured regularly with a digital caliper and tumor volumes were determined by the formula: π/6 × a × b2, (a = longest diameter, b = shortest diameter). In addition, NMRI^nu/nu^ mice were analyzed by positron emission tomography (PET) and magnetic resonance tomography (MRT) at defined time points as reported below. The animals were sacrificed when the recurrent tumor reached the mean diameter of 12–15 mm and cryogel scaffold were collected and analyzed via fluorescence microscopy as described above.

To analyze the *in vivo* anti-tumor efficacy of the artificial therapeutic device, either empty scaffolds or cryogels housing bsAb-releasing MSCs (5 × 10^5^) were s.c. implanted into the right legs of NMRI^nu/nu^ mice distinguished as control (n = 5) or treatment group (n = 5) respectively. Following four days, a freshly prepared cell mixture containing 1.5 × 10^6^ human T cells and 5 × 10^5^ MOLM-13-Luc^+^ cells was s.c. injected into the left legs of all mice. All experimental mice were analyzed via optical imaging (see below) on day 0, 1, 4 and 5.

To assess plasma concentration levels of bsAb, six NMRI^nu/nu^ mice were s.c. transplanted with cryogels housing bsAb-releasing MSCs (5 × 10^5^), whereas three NMRI^nu/nu^ mice received empty scaffolds (control mice). On the fourth day post-transplant, plasma was collected from blood samples obtained via cardiac puncture of anesthetized mice, subsequently euthanized according to local ethical committee guidelines. Thereafter, bsAb was purified by Ni-NTA affinity chromatography via the C-terminal his-tag and plasma concentration was evaluated via ELISA as previously described^22^.

### Positron emission tomography (PET) scans

PET analysis was performed as described elsewhere^27^,^28^,^29^,^30^,^31^. Briefly, for PET analysis NMRI^nu/nu^ mice were anesthetized using 9% ± 1% desflurane in 30% oxygen and placed on a heat mat. The anesthetized animals were localized in a prone position in axial direction of the scanner. A needle catheter was installed in a lateral tail vein for injection using a syringe pump. Positron emission tomography (PET) studies were performed with dedicated small animal PETs, NanoPET/CT (Mediso, Budapest, Hungary) and microPET(R) P4 (Siemens Medical Solutions, Erlangen, Germany). Transmission correction was performed with the attenuation CT or transmission scans of 10 min using a ^57^Co point source that were performed before tracer application. Data were acquired over 60 min. Simultaneously with the start of data acquisition, 4–8 MBq fluorodeoxyglucose ([^18^F] FDG) (Helmholtz-Zentrum Dresden-Rossendorf, HZDR) were injected in the animals within 1 min. PET images were iteratively reconstructed by a three-dimensional ordered-subset expectation maximization algorithm (3D OSEM/MAP) with transmission correction and with voxel size of 0.050 × 0.050 × 0.050 cm. Three-dimensional tumor regions of interest (ROI) were determined for subsequent data analysis. The standardized uptake values (SUV, g/mL) and standardized uptake ratios (SUR, as ratio of the SUVs of the tissue of interest and the blood SUV, derived from a region over the caudal arteria abdominalis and vena cava) were used to quantify the [^18^F] FDG uptake and the respective kinetics were determined using ROVER software (ROI Visualisation, Evaluation and Image Registration, ABX Radeberg, Germany). The metabolic trapping rates (Km) were calculated using irreversible two-compartment model^32^ based on the activity concentration blood/tissue curves.

### Magnetic resonance tomography (MRT) analysis

For MRT analysis mice were anesthetized as described above and positioned in a warmed cradle. A 30 cm horizontal bore Bruker Biospin magnet operating at 7 Tesla (BioSpec 70/30 USR, Bruker BioSpin GmbH, Karlsruhe, Germany), was used with a S116 gradient set to perform all MRT experiments. An echo planar imaging (EPI) transceiver 1 H 50 W coil with a 38.0 mm inner diameter was used for signal transmission and detection. RARE sequence (Rapid Acquisition with Relaxation Enhancement) anatomical imaging (FOV = 8.00 cm, SI = 0.75/0.75 mm, MTX = 384, TR = 5643 ms, TE = 36.7, FA = 180.0 deg, TA = 18 min, Echo = 1/1) was used to visualize the scaffold and the tumor.

### Optical imaging of tumor xenograft models

Luminescence imaging was performed using a dedicated small animal multimodal imaging system (Xtreme, Bruker, Germany) 10 min after intraperitoneal (i.p.) injection of 200 μl of D-luciferin potassium salt (15 mg/ml) (Thermofisher, Dreieich, Germany). Optical imaging was collected with a 1-minute exposure and pseudo color representations of light intensity were superimposed over the reference image. To quantify the detected light, a region of interest was manually selected over the signal intensity and the light emitted from each region was evaluated and quantified after background subtraction. In parallel, an X-ray photograph was taken from the animals at the same position.

### Statistical analysis

One-way analysis of variance (ANOVA) with Bonferroni Multiple Comparison test was used for statistical significance when multiple groups were compared, whereas Student’s *t*-test was used to detect significant differences between two groups. Statistical analysis was performed using GraphPad Prism Software (La Jolla, CA, USA). (***p < 0.001, **p < 0.01, *p < 0.05).

## Results

### Development and characterization of starPEG-heparin cryogel matrix

**Figure 2:**
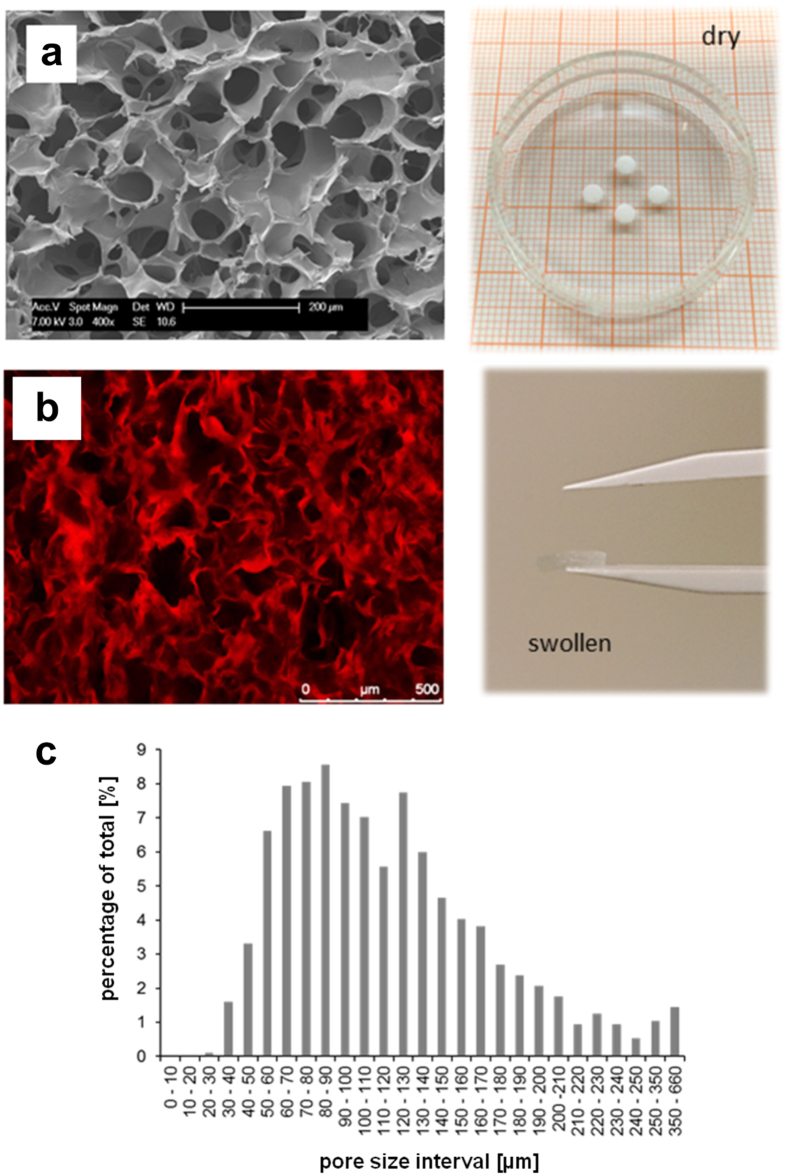
Characteristics of starPEG-heparin cryogels. (**a**) Scanning electron microscopy (SEM) analysis of the modular starPEG-heparin cryogel structure produced via cryogelation technology by chemically cross-linking of EDC/sulfo-NHS-activated carboxyl groups and amine end-functionalized star-PEG. SEM image (left) and digital image (right) of the cryogel scaffold in the dry state are reported. (**b**) Confocal laser scanning microscopy cLSM image (left image) and digital image (right image) of Alexa fluor647-labelled cryogel scaffold after swelling in PBS. (**c**) The pore size was quantified with cLSM images (n = 970 measurements were made for pore size analysis).

Macroporous starPEG-heparin cryogels were prepared by combining hydrogel network formation via chemical cross-linking of starPEG and heparin (molar ratio 1.5) with the cryogelation technology as previously described^7^,^8^,^9^. The unique porous and spongy structure of the dry or PBS-swollen starPEG-heparin material (bulk stiffness: 3 ± 2 kPa, strut stiffness: 101 ± 45 kPa) analyzed via scanning electron microscopy (SEM) or confocal laser scanning microscopy (CLSM) respectively (Fig. 2a and b) revealed a sophisticated macrostructure characterized by interconnected macropores mostly in the range of 20 to 250 μm (Fig. 2c), corresponding to optimal size range supporting the ingrowth of the majority of mature cell types^33^. To improve integrin-mediated cell adhesion, the cryogel scaffolds (diameter: 6 mm, height: 3 mm) were functionalized with RGD (Arg-Gly-Asp)-containing peptide sequences, which in combination with the high surface-to-volume ratio offered by the artificial matrix ensures a rapid cell adhesion and a proper spreading of cells in the three-dimensional (3D) scaffolds.

### Cytocompatibility analysis of the cryogel-based therapeutic device

**Figure 3:**
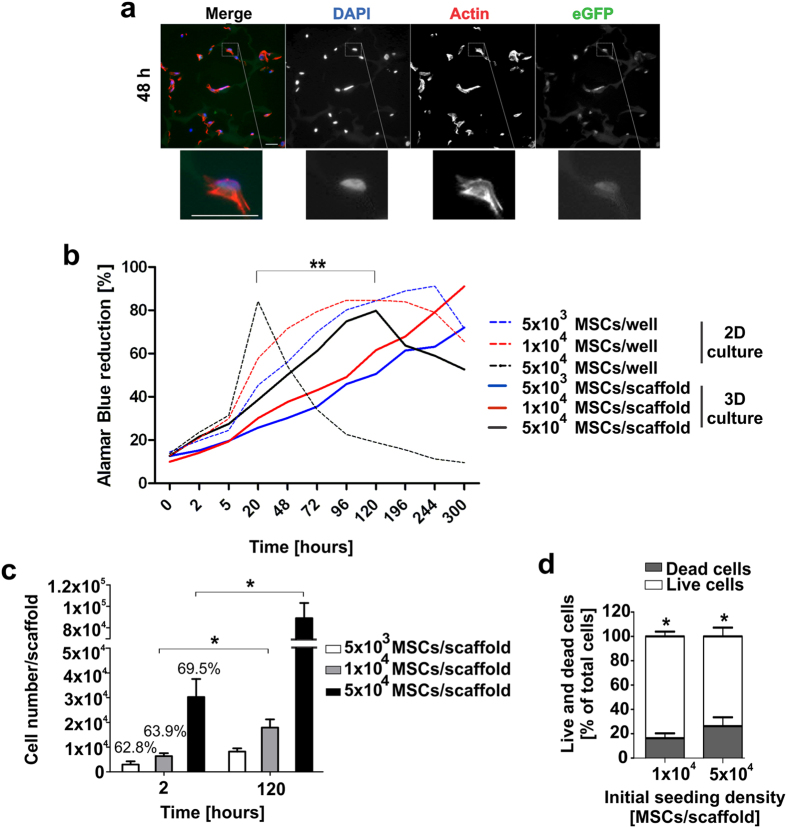
Analysis of cell-supporting properties of the starPEG-heparin cryogel. (**a**) Fluorescence microscopy images of 48 h-cultured MSCs in the cryogel scaffold seeded at initial cell number of 5 × 10^4^. Sections were counterstained with DAPI (blue) to detect nuclei, with Alexa fluor647 Phalloidin (red) for actin filaments, and visualized for eGFP expression cassette (green). Zoomed-in images of the inset show a bsAb-releasing MSC adhered to the components of the starPEG-heparin matrix. Scale bars, 30 μm. (**b**) Metabolically active gene-modified MSCs seeded in 96-well plates in cryogel scaffolds (3D) and in 2D in parallel were analyzed via Alamar blue assay. The proliferative cell activity is expressed as the percentage of Alamar blue reduction over time for each initial cell number used. Dashed lines correspond to MSCs seeded in 2D. Data are shown as the means of three independent experiments performed in triplicates. (**c**) Total cell numbers of bsAb-releasing MSCs on cryogel scaffolds was calculated 2 h and 120 h after initial cell seeding by measuring the optical density of Alamar blue reduced by the cryogel-housed MSCs at reported time points. The absorbance measured in 2D assays with equivalent number of cells was used as reference value for total available cells to determine the efficiency of initial cell seeding. By setting the seeding efficiency of 2D-seeded MSCs to 100%, relative percentages corresponding to 3D samples were calculated accordingly and are reported above 2 h columns. Data are shown as the means ± SD of three independent experiments. (**d**) Fractions of live (TUNEL^-^) and dead (TUNEL^+^) MSCs detected after 10 days of cultivation in the scaffolds are reported for two different initial cell numbers. Data show the means ± SD of three independent experiments performed in triplicates. Statistical significance was determined using Student’s *t*-test. *p < 0.05; **p < 0.01.

To assess the capability of the starPEG-heparin cryogel in supporting the ingrowth and the proliferation potential of a cell-based delivery system of immunoagents, cytocompatibility analysis were performed by housing a gene-modified human bone marrow-derived MSCs cell line (SCP-1 cells) in the cryogel material over prolonged *in vitro* cultivation time. The proliferation rate of the recently established CD33-CD3 bsAb-releasing MSCs^21^,^22^,^26^ was at first verified via fluorescence microscopy analysis. Therefore, varying numbers of bsAb-secreting MSCs were cultivated in cryogel matrices functionalized with 80 μg/ml RGD-containing peptides and following 48 h, 96 h and 120 h of culture, cryosections of MSCs/cryogel samples were collected and stained to visualize the distribution of viable cells along the scaffold’s pores. Analysis of stained cryosections revealed that modified MSCs increased in number starting from 48 h of cultivation to 120 h (Supplementary Fig. 1), independently on the cell seeding density used. In addition, already after 48 h of incubation, modified MSCs were clearly detectable within the cryogel via their characteristic spindle-like morphology and displayed good cell adherence, homogeneous cell distribution along the macropores and a remarkable fit to the matrix (Fig. 3a). The proliferative activity of modified MSCs detected within the cryogel scaffolds was subsequently quantified via Alamar blue assay. The increasing reduction of Alamar blue dye (in percentage) detected over 120 h of *in vitro* cultivation reflecting metabolically active cells confirmed that the number of modified MSCs along the macropores increased proportionally with prolonged cultivation time (Fig. 3b). In addition, comparison of the optical density of the Alamar blue reduced by 3D-seeded MSCs to that of equivalent numbers of MSCs seeded in 96-well plates as conventional two-dimensional (2D) cultures (control), showed that the seeding efficiency of MSCs in 3D was approximately 70% for each of the three different initial cell numbers used (Fig. 3c). The doubling of the total cell number detected 120 h after initial cell seeding, further confirmed the significant proliferation of MSCs in the macropores of the starPEG-heparin cryogels.

Moreover, evaluation of the apoptotic index of MSCs housed in the cryogel scaffold via TUNEL assays revealed that after ten days of 3D culture nearly 80% of MSCs could be distinguished as living cells (TUNEL^−^ cells) (Fig. 3d and Supplementary Fig. 2), underlining the remarkable cytocompatibility of this artificial device and its capacity in supporting MSCs proliferation and survival over prolonged cultivation time.

### Cell adherence of bsAb-releasing cells to the cryogel scaffold

Considering the importance for future clinical application to avoid cell loss and to maintain MSCs proliferation confined at the scaffold site, the adherence of gene-modified MSCs to the cryogel matrix was tested in a first step by colony formation assays.

When cryogel-housed MSCs (3D samples) were cultivated for 5 or 10 days in 96-well plates almost no MSCs loss could be detected at the cavity walls by crystal violet staining for all initial cell numbers used, whereas conventional 2D MSCs cultures performed in parallel as a control reported significant staining (Supplementary Fig. 3a). These results were underpinned by measuring the optical density of the dissolved crystal violet dye. The values obtained for the cavities that had housed the 3D samples were similar to the values of the negative control characterized by cell culture medium only and extremely low in comparison to the cavities housing conventional 2D MSCs cultures (Supplementary Fig. 3b).

**Figure 4:**
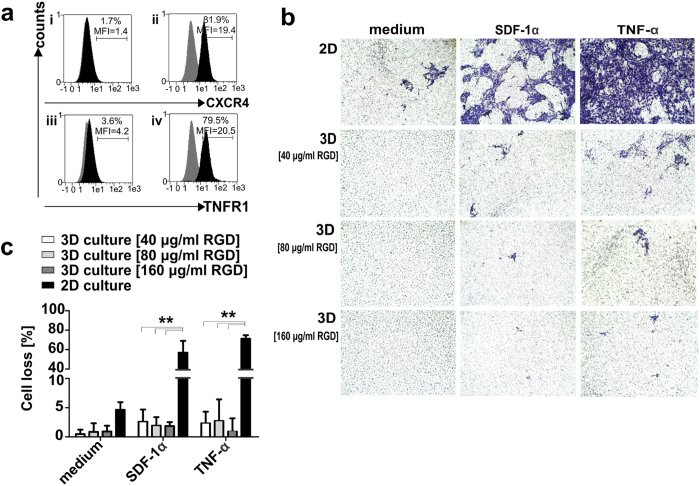
Migration rates of cryogel-housed modified MSCs towards stimuli. (**a**) Extracellular (i; iii) and intracellular (ii; iv) expression of CXCR4 and TNFR1 receptors on modified MSCs detected via flow cytometry upon staining with anti-CXCR4/PE and anti-TNFR1/PE mAbs (black), or matched isotype control Abs (grey). Fractions and the mean fluorescence intensity (MFI) of total cells are reported. (**b**) Induced and spontaneous migration (medium without chemoattractants) were examined either in 2D cell cultures or in cryogel-housed MSCs samples (3D) functionalized with 40 μg/ml, 80 μg/ml or 160 μg/ml RGD-containing peptides. Representative images are reported for MSCs migration rates in the presence or absence of chemoattractants for 2D and 3D cell cultures respectively. (**c**) Percentages of migrated cells are reported as the means ± SD of total cells detected in five random microscope-viewing fields for three independent experiments. Statistical significance was determined using one-way ANOVA with Bonferroni multiple comparison test. **p < 0.01.

Nevertheless, given the migratory potential of MSCs^34^,^35^,^36^, especially in response to MSC-specific chemoattractants, like stromal-derived factor-1α (SDF-1α)^36^,^37^ and the pro-inflammatory cytokine tumor necrosis factor-alpha (TNF-α), it was of main interest to further investigate the cell adhesion properties of the cryogel scaffold under *in vivo*-like conditions. The high expression levels of the receptors CXC chemokine receptor-4 (CXCR4) and tumor necrosis factor receptor-1 (TNFR1) confirmed a notable migratory potential of the modified MSCs used in this study towards these specific stimuli. In line with recent findings^37^,^38^, both CXCR4 and TNFR1 receptors were found predominantly expressed in the intracellular compartment of 81.9% and 79.5% modified MSCs respectively (Fig. 4a).

Therefore, *in vitro* transwell migration assays were performed in the presence of high concentrations of SDF-1α and TNF-α after seeding 1 × 10^5^ modified MSCs either in starPEG-heparin cryogels functionalized with different concentrations of RGD-containing peptides or as conventional 2D control cultures in parallel. After 20 h of *in vitro* cultivation no significant cell migration was observed from the 3D samples, whereas high numbers of migrating cells were detected for the control 2D cultures (Fig. 4b). Interestingly, the low rates of MSCs loss detected for cryogels functionalized with 40 μg/ml RGD peptides further decreased with increasing RGD peptide concentration. (Fig. 4c).

### *In vivo* characterization of the immunotherapeutic system

Importantly, the established system also showed remarkable *in vivo* biocompatibility, promoting survival and metabolic activity of MSCs for prolonged time, without provoking evident inflammatory response or scaffold rejection.

**Figure 5:**
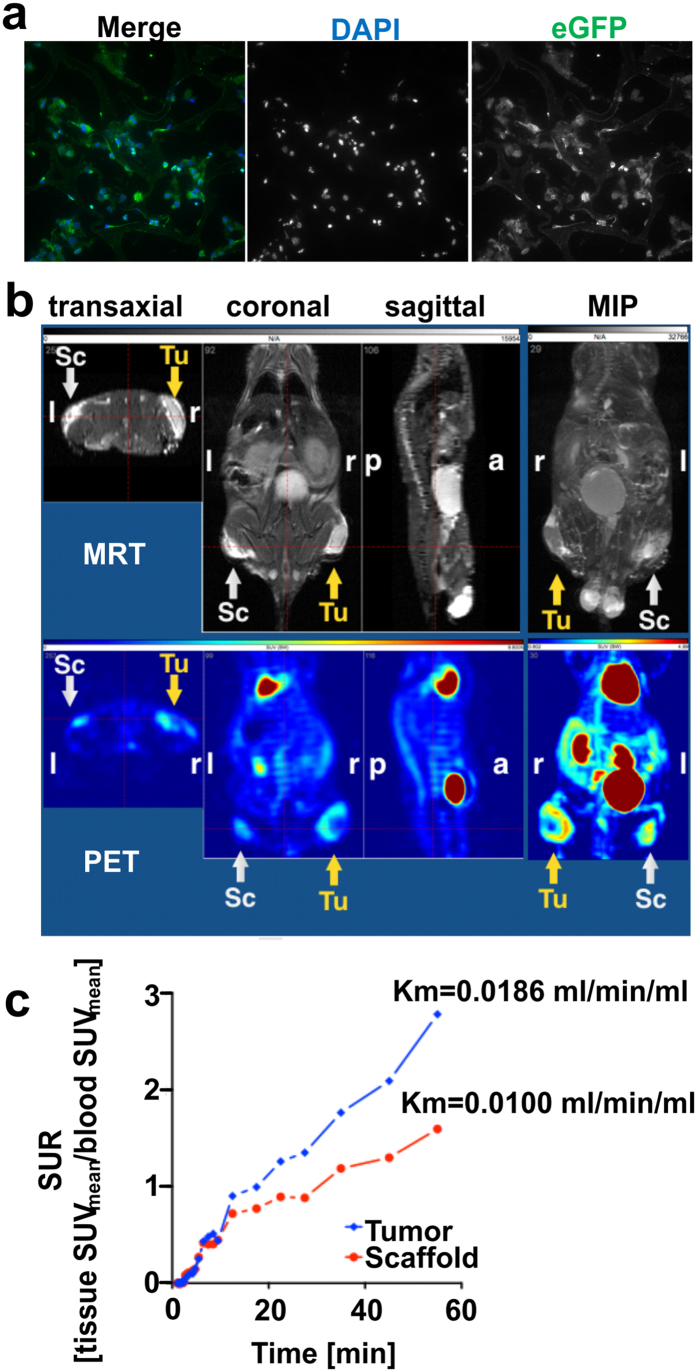
*In vivo* analysis of the cryogel-based therapeutic device. (**a**) Representative immunofluorescence staining of a 7 μm cryosection of a MSCs/cryogel sample performed upon its retrieval 13 days after implantation in NMRI^nu/nu^ mice. Nuclei were counterstained with DAPI (blue) and cytoplasm was visualized with an anti-eGFP mAb (green). Scale bar, 30 μm. (**b**) Magnetic resonance tomography (MRT) and Positron emission tomography (PET) orthogonal images are reported together with the relative maximum intensity projections (MIPs) for one representative mouse out of 22. Sc = scaffold, Tu = tumor, r = right, l = left, a = anterior, p = posterior. (**c**) [^18^F] FDG-time-activity curves of the PET study is shown for one representative mouse. The activity concentration is shown as standardized uptake ratio (SUR) for the Sc and the Tu at day 11 and the relative metabolic trapping rates (Km) reflecting the glucose consumption are reported.

Immunofluorescence analysis of cryogel-housed MSCs performed upon retrieval of the scaffolds from their subcutaneous implantation in immunodeficient NMRI^nu/nu^ mice showed high rates of living MSCs (Fig. 5a) and no evidence of infiltrating immune cells could be detected at the implantation site after 13 days of transplantation (data not shown).

Additionally, analysis of implanted cell-seeded scaffolds via both anatomical magnetic resonance tomography (MRT) and positron emission tomography (PET) based on the uptake of fluorodeoxyglucose ([^18^F] FDG)^27^ (Fig. 5b) revealed that the time-activity curves of [^18^F] FDG uptake by cryogel-housed MSCs increased in the late phase (Fig. 5c) for both modified MSCs and s.c. injected CD33^+^ MOLM-13 cells, an AML model cell line used as metabolically active control cells and the metabolic activity of cryogel-housed MSCs and tumor cells followed comparable trends overtime (Supplementary Fig. 4). The evidence that the metabolic volume of the scaffolds did not significantly change, whereas the glucose uptake by the MSCs increased with a doubling time of 2.4 days, reaching 60–90% of tumor [^18^F] FDG uptake, clearly indicated that MSCs number increased overtime and an efficient cell proliferation occurred within the scaffold also *in vivo*.

### *In vitro* effectiveness of the biohybrid bsAb-releasing device

**Figure 6:**
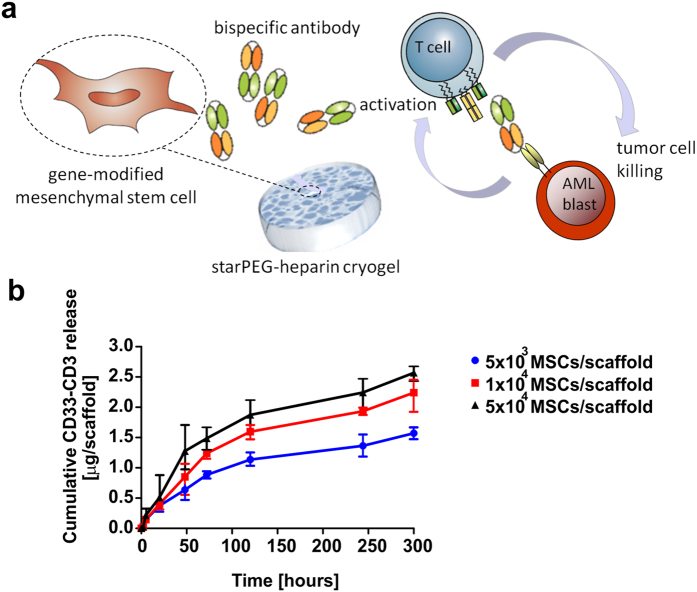
Quantitative analysis of the released bsAb CD33-CD3 by cryogel-housed MSCs. (**a**) Representative illustration of the cryogel-housed scBsAb-releasing MSCs system. Genetically modified MSCs were confined in the starPEG-heparin cryogels for a constant release of the bsAb CD33-CD3 suitable for the redirection of T-cells to AML target cells over prolonged time. (**b**) The amount of bsAb CD33-CD3 produced by modified MSCs seeded at reported seeding densities in the cryogel scaffold was quantified by ELISA. The antibody concentration (reported in μg) released in the cell culture supernatants was determined at different cultivation time points for an overall time of 300 h. The cumulative release of the bsAb detected over time is reported as the means ± SD of three independent experiments.

*In vitro* studies provided first proof-of-principle of the effectiveness of the established cryogel-based platform in efficiently retargeting T-lymphocytes towards AML blasts (Fig. 6a). Quantification of the bsAb released *in vitro* by modified MSCs housed in the cryogel scaffold for an overall time of 300 h revealed that the transplantable MSC/cryogel device displayed remarkable capacity to constantly secrete sustained amounts of highly efficient bsAb over prolonged times (Fig. 6b). Detectable bsAb concentrations were found in the *in vitro* cell culture supernatant (200 μl) already a few hours after cell seeding by enzyme-linked immunosorbent assays (ELISA) via detection of an oligo-His-Tag at the C-terminus of the bsAb. Within the first 48 h high amounts of bsAb were released by the therapeutic device, ranging from 0.6 to 1.3 μg/scaffold, depending on the initial cell seeding density. According to our previously performed *in vitro* and *in vivo* studies^14^,^22^,^23^, the detected bsAb amount was considered to be sufficient to exert an effective anti-tumor response.

**Figure 7:**
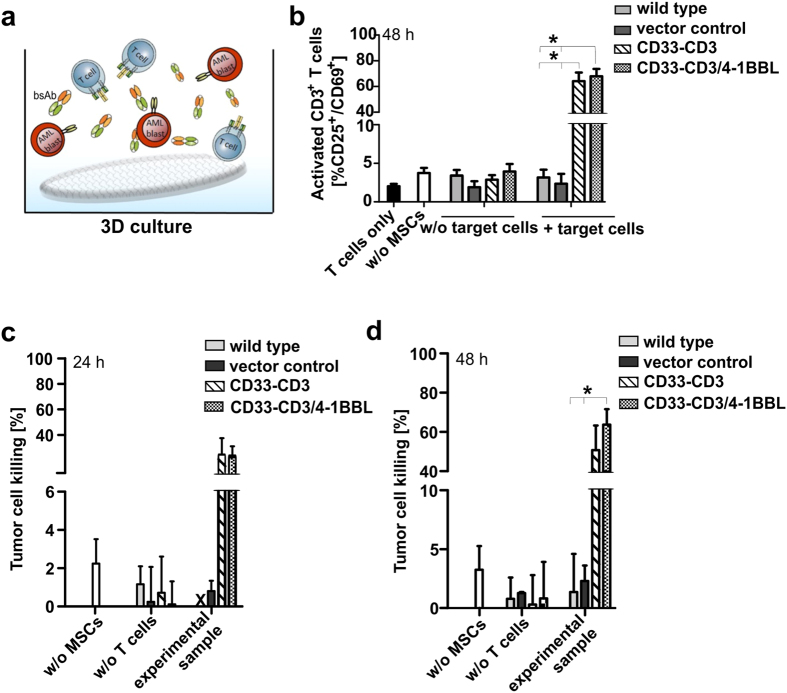
*In vitro* effectiveness of the bsAb-releasing MSCs/cryogel system. (**a**) A schematic representation of the experimental settings established for the *in vitro* T-cell activation and tumor cell killing assays is reported. (**b**) The expression of T-cell-specific activation markers was determined by flow cytometry after 48 h of incubation of T-cells and 1 × 10^4^ CD33^+^ MOLM-13 cells at an effector-to-target (E:T) ratio of 1:1 in the presence or absence of 1 × 10^4^ genetically cryogel-housed MSCs. The activation status of redirected T cells is reported as the percentage of CD25^+^/CD69^+^ cells detected on the total of CD3^+^ T-cell number. (**c**,**d**) Specific lysis of ^51^Cr labeled CD33^+^ MOLM-13 cells was analyzed via standard chromium release assay after 24 h or 48 h of co-cultivation with 1 × 10^4^ T-cells at an E:T ratio of 1:1 in the presence or in the absence of 1 × 10^4^ bsAb-releasing and 4-1BBL-expressing modified MSCs seeded in the cryogel matrix. Data are reported as the means ± SD for three different T-cells donors. Statistical significance was determined using one-way ANOVA with Bonferroni multiple comparison test. *p < 0.05. X = not detectable.

Therefore, the therapeutic potential of the MSCs/cryogel device was first experimentally assessed via functional *in vitro* studies. The efficient retargeting of human CD3^+^ T-lymphocytes towards AML blasts triggered by the bsAb-releasing device was detected via co-culture experiments with CD33^+^ MOLM-13 cells, and human T-cells at a low effector-to-target (E:T) cell ratio of 1:1 (Fig. 7a). Flow cytometry analysis performed after 48 h of co-incubation revealed that nearly 70% of CD3^+^ T-cells expressed the specific activation markers CD25^+^ and CD69^+^ on their cell surface upon cross-linkage with tumor cells via MSC-released bsAb in both 3D samples (Fig. 7b) and in 2D samples performed in parallel as a control (Supplementary Fig. 5a). As a result, notable CD33^+^ target cell killing was detected via standard chromium release cytotoxicity assays already after 24 h (Fig. 7c), leading to approximately 50% to 70% of tumor cell lysis after 48 h of incubation in the presence of bsAb-releasing or bsAb-releasing/4-1BBL-expressing MSCs respectively (Fig. 7d). According to our previously published data^14^,^22^, the presence of the additional T-cell co-stimulatory molecule 4-1BBL (CD137L) resulted in an amplification of the T-cell-mediated tumor cell killing via the 4-1BB/4-1BBL signaling pathway in comparison to the stimulus provided by the CD33-CD3 bsAb alone. This more pronounced tumor-specific cytotoxicity effect detected in the presence of the bsAb-producing MSCs further modified for the ectopic expression of the 4-1BB ligand resulted to be similar to the anti-tumor effect observed in functional *in vitro* studies performed in parallel under the same experimental conditions as conventional control 2D samples (Supplementary Fig. 5b and c), suggesting that T-cells can be equally and efficiently redirected against tumor cells in both systems.

### *In vivo* anti-tumor efficacy of the MSCs-based therapeutic organoid

To demonstrate that the artificial device could trigger detectable anti-tumor effects also *in vivo*, additional studies were conducted in immunodeficient mice.

**Figure 8:**
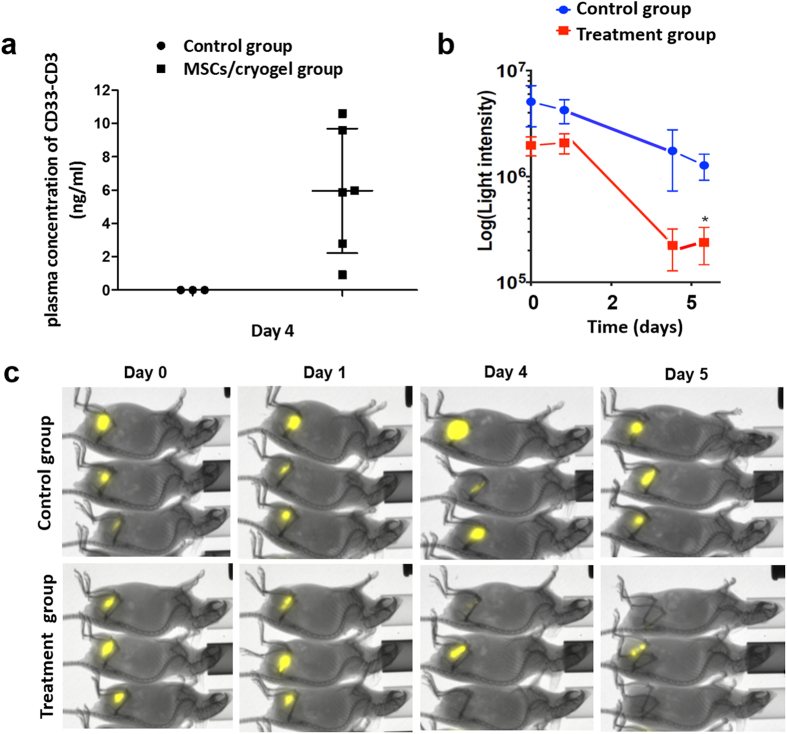
*In vivo* anti-tumor efficacy of the MSCs/cryogel therapeutic device. (**a**) Plasma concentration of bsAb CD33-CD3 was detected in NMRI^nu/nu^ mice, s.c. transplanted with cryogels housing 5 × 10^5^ bsAb-releasing MSCs (MSCs/cryogel group) or empty scaffold (control group). The plasma level of the immunoagent was detected and quantified via ELISA on the fourth day post-transplantation. Data are shown as mean ± SD of six or three experimental mice. (**b**) Immunodeficient NMRI^nu/nu^ mice were s.c. transplanted with cryogel-housed MSCs (n = 5) or empty scaffolds (n = 5) into the right legs and distinguished as treatment and control group respectively. Following 4 days, 1.5 × 10^6^ human T-cells were mixed with 5 × 10^5^ CD33^+^ MOLM-13 expressing firefly luciferase and s.c. injected into the left leg of both mice groups. Luminescence imaging of anesthetized mice was performed 10 min after i.p. injection of 200 μl of D-luciferin potassium salt starting at day 0, followed at day 1, day 4, and day 5 and the persistence of viable MOLM-13-Luc^+^ evaluated over the experimental time in both mice groups is reported as light intensity. Data represents the mean ± SD of 5 mice per group starting from day 0 to day 5. Statistical significance was determined using Student’s *t*-test. *p < 0.05. (**c**) Representative optical imaging of 3 out of 5 mice from the control and the treatment group are reported respectively.

In a first step, plasma levels of bsAb CD33-CD3 were evaluated in order to verify that cryogel-housed MSCs can release sustained amounts of bsAb also *in vivo.* Therefore, NMRI^nu/nu^ mice were s.c transplanted with the therapeutic device containing 5 × 10^5^ bsAb-releasing MSCs or empty scaffolds and used as reference mice for analyzing the bsAb concentration yielded at plasma level at an established time point. Thus, on the fourth day post-transplantation the plasma concentration of bsAb was quantified by ELISA. As reported in Fig. 8a, detectable concentrations of bsAb ranging from 0.9 ng/ml to 10.4 ng/ml could be measured in the plasma of MSCs/cryogel transplanted mice. These data show that the bsab released from the cryogel system reaches the circulation. Moreover, the estimated concentration of bsAb in peripheral blood of experimental mice is in the range of previously determined EC_10_ to EC_50_ values^14^.

Therefore, the same number of cryogel-housed bsAb-releasing MSCs were s.c. implanted in the right legs of NMRI^nu/nu^ mice (treatment group), whereas control NMRI^nu/nu^ mice received empty scaffolds (control group). After four days, all immunodeficient mice were s.c injected with human T-cells and MOLM-13-Luc^+^ cells at an E:T ratio of 3:1 in their left legs. Analysis of the luciferase activity of CD33^+^ tumor cells was performed for all mice in parallel for an overall time of 5 days. While the luciferase activity of MOLM-13-Luc^+^ cells could easily be detected in the control group over the whole experimental time, in all treated mice the signal intensity decreased overtime (Fig. 8b and c) and in most of the treated mice it was no more detectable already from days 4 to 5 (Fig. 8c). Not unexpected, due to the allogeneic setting, to some extend the allogeneic T cells also attacked the tumor cells, but the decrease of tumor cells in the treated group was statistically significant (Fig. 8b). In summary, these results underline that under our experimental conditions the bsAbs released *in vivo* by cryogel-housed MSCs (i) are able to reach the blood stream, (ii) are fully functional in retargeting of T cells, and (iii) reach sufficient concentrations to exert an effective and detectable anti-tumor response.

## Discussion

The increasing interest regarding biocompatible materials in the field of immunotherapy of tumors has led to the production of a great variety of biohybrid systems aimed to improve the anti-tumor effectiveness of small immunoagents. In this context, a plethora of examples of cell-based delivery systems loaded into natural or synthetic scaffolds are described in literature [*e.g.* refs 19, 39, 40 and 41].

Not surprisingly, most of these artificial immune organs, recently named as “immunotherapeutic organoids”^20^, preferentially employ gene-modified MSCs as cell vehicle option for delivery immunotherapeutics, given their great expansion capacity, easiness of handling, genetic manipulation and especially low immunogenic properties^20^,^39^. In this context, interesting examples of this approach are represented by the study conducted by Compte and colleagues aimed to develop an anti-CEA diabody-releasing MSCs/matrigel system^39^ or by Eliopoulos *et al*. describing the use of IL-2 producing-MSCs artificial device for breast cancer treatment^42^. In a recent study we also could demonstrate the anti-tumor efficacy of human MSCs gene-modified for the production of bispecific antibodies in triggering significant anti-tumor responses with low risk of side effects^22^. Taking advantage from our previous achievements we decided to further optimize the method by developing for the first time a biomaterial-supported stem cell factory for customized sustained release of a bsAb for T cell-mediated AML immunotherapy as alternative approach to the continuous infusion of short-lived immunoagents for antigen-specific AML treatment.

Within the wide scenario of novel biomaterials developed during the last decades, the starPEG-heparin cryogel was chosen as therapeutic platform as it displays characteristic features that strongly meet the main requirements for developing a MSCs-based immunotherapeutic device. In comparison to other synthetic and natural scaffolds employed for developing ”living” bsAb delivery technologies^19^,^39^,^40^, the starPEG-heparin cryogel-supported stem cell factory offers several important advantages by combining the useful properties of the macroporous biomaterial with the benefits of bsAb-secreting MSCs.

This small and low-invasive sponge-like material results to be effective in guaranteeing a rapid and efficient transport of nutrients and therapeutic bsAbs via the interconnected macropores and given its capacity to withstand large deformation without losing integrity, the artificial therapeutic device ensures protection of housed cells from mechanical stress during the implantation and an easy retrieval once the therapeutic effect is achieved.

In addition, based on the fact that FDA-approved synthetic PEG polymers are currently accepted and some received already market approval for different applications given their low interaction with blood components and high biocompatibility^43^, the use of a PEG-based material resulted in a reliable choice for our experiments in view of potential clinical applications. The encouraging biocompatibility features observed in our preliminary *in vivo* studies suggest promising achievements in potential future settings. The evidence that the subcutaneous implant is well tolerated *in vivo* and no relevant inflammatory events or scaffold capsulation or rejection could be detected in transplanted mice over the experimental time encourage further *in vivo* studies and bodes similar tolerogenicity in humans as well.

Moreover, the underlying biomimetic starPEG-heparin network allows for many different biomolecular functionalization schemes useful to possibly improve the interaction of therapeutic cell-based delivery systems to the biohybrid support if needed^7^. In line with the observation reported by other groups [*e.g.* refs 44, 45], RGD-functionalization of the cryogel support was already sufficient to dramatically reduce cell loss and to prevent the escape of MSCs even under unfavorable conditions, without impairing cell motility and distribution within the scaffold’s pores. Most importantly, proliferation and survival of the genetically modified MSCs in this artificial microenvironment resulted in a continous production of the bsAb CD33-CD3 including *in vitro* and even experimental mice.

The high *in vitro* T cell-mediated anti-tumor effect observed already at early time points and in the presence of low effector to target cell ratios underlined the effectiveness of the artificial device. In addition, the observation that the ectopic expression of a specific T cell co-stimulatory molecule 4-1BBL on MSCs surface acts in concert with the bsAb stimulus is in line with our recently published data^14^,^22^. Although the effect of the co-stimulus seems to be still weakly recognizable at 48 h it is important to mention that the 4-1BBL stimulus, essential for long-lasting memory T cell response, is known to influence T cell activity in late primary responses, following a previous activation signal provided by the CD3 complex signaling^46^,^47^. Therefore, we believe that the effect of the co-stimulus on T cell mediated-anti-tumor response would have been more likely appreciated in late experimental time points, but unfortunately due to the limited *in vitro* conditions the experimental readout could not be extended further. Therefore, it will be interesting to verify whether or not the synergistic activity of the 4-1BBL co-stimulus improves the T cell response *in vivo* in comparison to the stimulus provided by the bsAb alone and to which extent.

Considering the highest density of cells used in our *in vitro* experiments (5 × 10^4^ MSCs/cryogel) the cumulative amount of recombinant protein released by the cryogel system was evaluated to range from 1.1 μg to 1.6 μg within the first 48 h of cultivation. According to our experimental conditions, approximately 32 pg/cell as minimum can be produced by the immunotherapeutic device system. In previous studies, we showed that the administration of 10 μg of the same but recombinantly expressed bsAb CD33-CD3 led to an eradication of over 90% of CD33^+^ tumor cells in the bone marrow of immunodeficient mice with established AML tumor^23^. Accordingly, we estimated that around 5 × 10^5^ gene-modified MSCs per cryogel could be sufficient to exert similar anti-tumor responses in mouse models, which also appeared to be a feasible cell number to be transplanted *in vivo* based on the applications reported by other groups for different MSCs-based therapeutic organoids^39^,^48^.

Indeed, when scafolds containing 5 × 10^5^ gene-modified MSCs were s.c. transplanted bsabs were released from the cryogel system and reached the circulation at concentrations in the range of previously estimated EC_10_ to EC_50_ values^14^,^23^. Moreover, the amount of released bsAbs was sufficient to efficiently redirect T cells to CD33 positive AML cells.

All together our data could demonstrate the feasibility and safety of housing gene-modified MSCs in a small compact PEG-heparin cryogel system for the constant production of bsAbs with a relevant *in vitro* and *in vivo* anti-tumor effectiveness. This study provides a basis for future applications of cryogel-housed therapeutic MSCs as a safe and effective delivery system of biologicals, paving the way to a novel and promising alternative approach for the treatment of both solid and blood tumors. By modifying MSCs for the production of different bsAbs, the cryogel-supported stem cell factory could be indeed easily customized for the treatment of various cancer types as well as for tumors expressing different TAAs according to the progression of the tumor stage^49^, resulting in an efficient, specific and personalized treatment.

## Additional Information

**How to cite this article**: Aliperta, R. *et al*. Cryogel-supported stem cell factory for customized sustained release of bispecific antibodies for cancer immunotherapy. *Sci. Rep.* **7**, 42855; doi: 10.1038/srep42855 (2017).

**Publisher's note:** Springer Nature remains neutral with regard to jurisdictional claims in published maps and institutional affiliations.

## Supplementary Material

Supplementary Figures

## Figures and Tables

**Figure 1:**
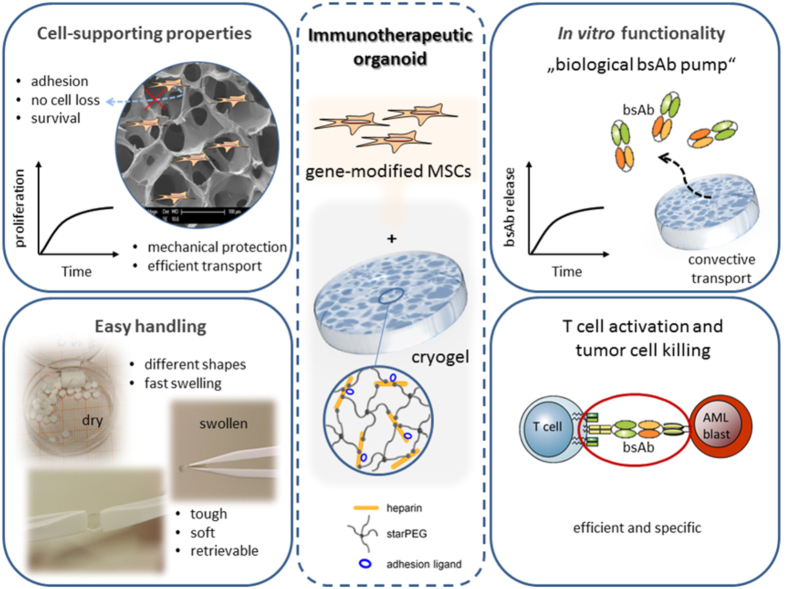
Scheme and properties of the cryogel-supported stem cell factory model designed for a customized substantial release of bispecific antibodies (bsAbs) for cancer immunotherapy. The starPEG-heparin cryogel scaffold displays outstanding biomolecular and mechanical features allowing the establishment of a cell-supporting microenvironment (left). By housing mesenchymal stromal cells (MSCs) genetically modified for the production of therapeutic bsAbs in the gel system functionalized with RGD peptides, the development of an immunotherapeutic organoid can be accomplished (middle). The artificial “biological bsAb pump” enables efficient and specific T-cell activation and tumor cell killing (right).
