# Supplementary material for: Cryogel-supported stem cell factory for customized sustained release of bispecific antibodies for cancer immunotherapy
Source: Sci Rep. 2017 Feb 16;7:42855. doi: 10.1038/srep42855 (PMC5311951; doi:10.1038/srep42855)
Supplement: Supplementary Figures [file srep42855-s1.doc]

Cryogel-supported stem cell factory for customized sustained release of bispecific antibodies for cancer immunotherapy

Roberta Aliperta^1^, Petra B. Welzel^2*^, Ralf Bergmann^1*^, Uwe Freudenberg^2^, Nicole Berndt^1^, Anja Feldmann^1^, Claudia Arndt^1^, Stefanie Koristka^1^, Marcello Stanzione^3^, Marc Cartellieri^4,5^, Armin Ehninger^6^, Gerhard Ehninger^8-10^, Carsten Werner^2,7^, Jens Pietzsch^1,11^, Jörg Steinbach^1,11^, Martin Bornhäuser^7-10^, Michael P. Bachmann^1,7,9-10#^

^1^Helmholtz-Zentrum Dresden Rossendorf (HZDR), Institute of Radiopharmaceutical

Cancer Research, Bautzner Landstrasse 400, 01328 Dresden, Germany.

^2^Leibniz Institute of Polymer Research Dresden (IPF), Hohe Strasse 6, 01069 Dresden, Germany.

^3^Institute of Physiological Chemistry, Technische Universität Dresden, Fetscherstrasse 74, 01307 Dresden, Germany.

^4^Cellex Patient Treatment GmbH, Tatzberg 47, 01307 Dresden, Germany.

^5^University Cancer Center (UCC), Technische Universität Dresden, Tumorimmunology, Fetscherstrasse 74, 01307 Dresden, Germany.

^6^GEMoaB Monoclonals GmbH, Tatzberg 47, 01307 Dresden, Germany.

^7^DFG-Center for Regenerative Therapies Dresden, Technische Universität Dresden, Fetscherstrasse 105, 01307 Dresden, Germany.

^8^Medical Clinic and Policlinic I, University Hospital 'Carl Gustav Carus', Technische Universität Dresden, Fetscherstrasse 74, 01307 Dresden, Germany.

^9^DKTK (German consortium for Translational Cancer Research), Dresden, Germany National ^10^National Center for Tumor Diseases (NCT), Dresden, ‘Carl Gustav Carus’ TU Dresden, Dresden, Germany.

^11^Department of Chemistry and Food Chemistry, School of Science, ‘Carl Gustav Carus’ TU Dresden, Germany

*contributed eqally

^#^To whom the correspondence should be addressed:

Helmholtz-Zentrum Dresden-Rossendorf (HZDR),

Institute of Radiopharmaceutical Cancer Research

Postbox 51 01 19

Bautzner Landstrasse 400

01328 Dresden, Germany.

University Cancer Center (UCC) Carl Gustav Carus Technische Universität - Dresden

Tumorimmunology

Fetscherstrasse 74

01307 Dresden, Germany.

Tel. HZDR : 0049 351 260 3170

Tel. UCC, Tumorimmunology: 0049 351 458 2101/ 0049 351 458 4177

m.bachmann@hzdr.de

**Supplementary Fig. 1**
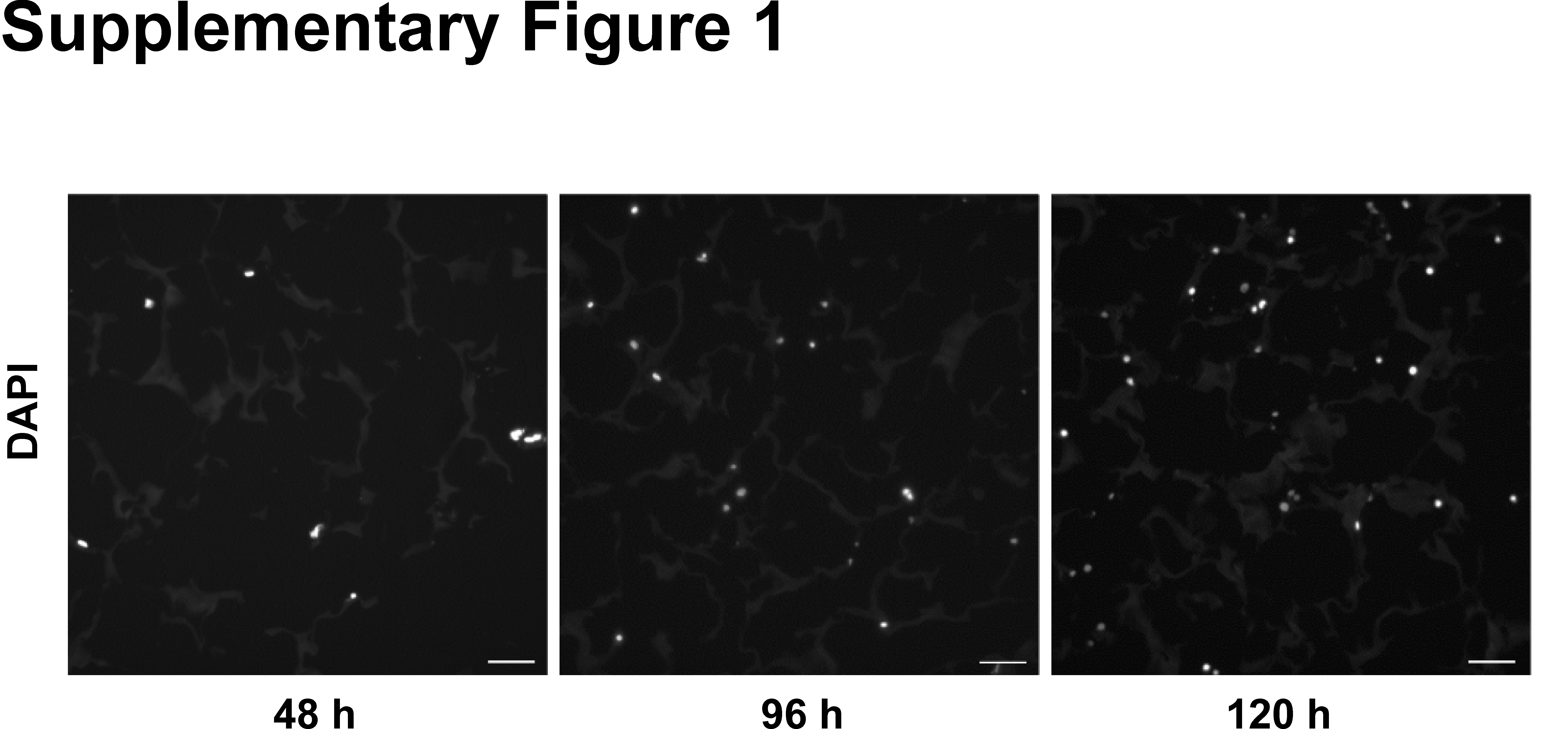


**Supplementary Fig. 1.** Proliferation rates of bsAb-releasing MSCs housed in cryogel scaffolds. Modified MSCs were cultivated at varying seeding numbers on cryogels scaffold for an overall time of 120 h. Representative fluorescence microscopy images of proliferating MSCs after 48 h, 96 h and 120 h of culture is reported for the initial seeding density of 1x10^4^ MSCs/scaffold. Cryosections of the MSCs/cryogel system were counterstained with DAPI to visualize nuclei. Scale bars, 30 μm.

**Supplementary Fig. 2**


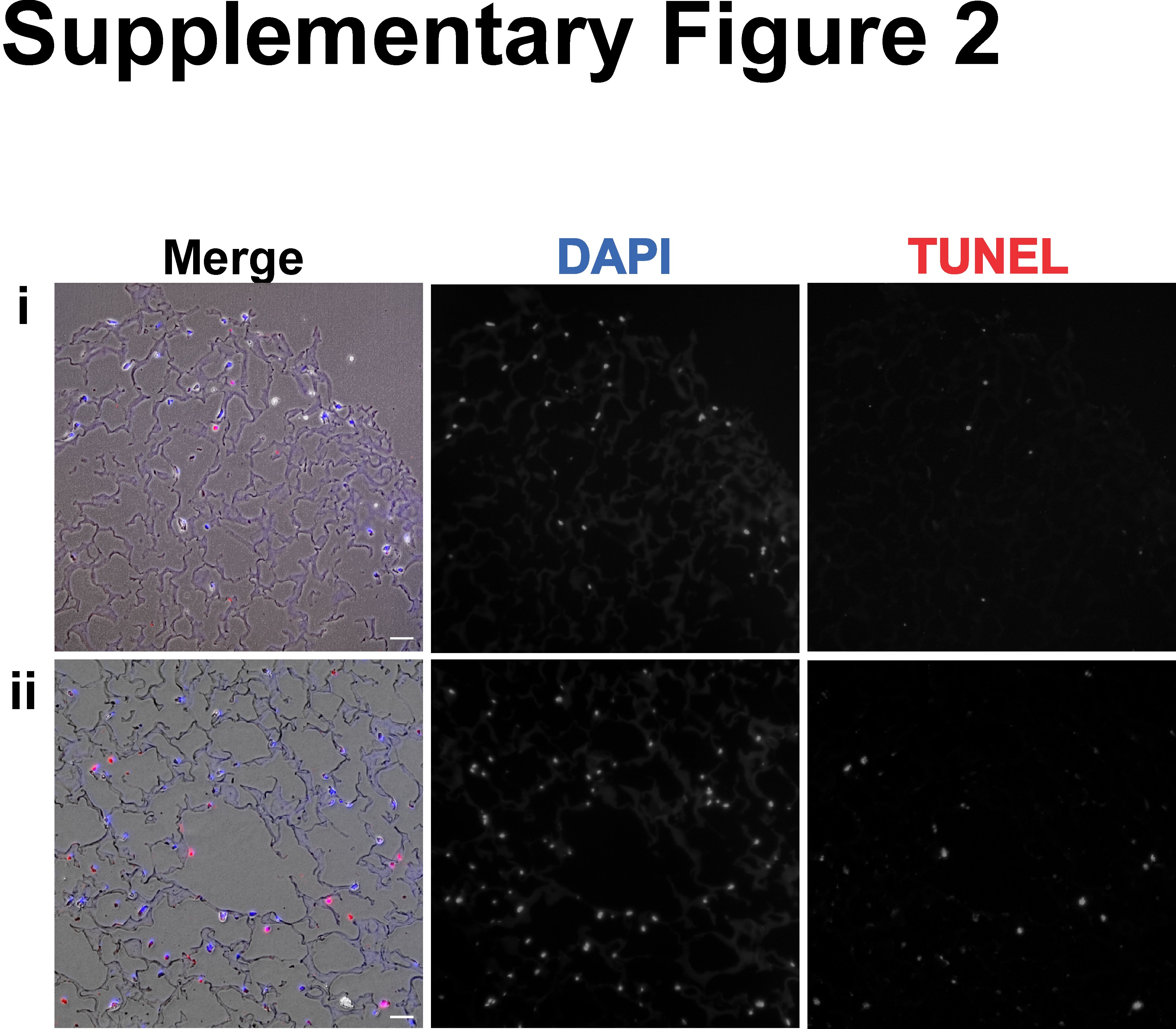


**Supplementary Fig. 2.** Cell viability of MSCs cultured in starPEG-heparin cryogels. After 10 days of cultivation, the viability of (i) 1x10^4^ or (ii) 5x10^4^ modified MSCs seeded in the cryogel scaffolds was determined via TUNEL assay by staining cryosections of MSCs/cryogel samples with TUNEL reaction mixture to identify apoptotic cells (red). Nuclei (blue) were counterstained with DAPI. Scale bars, 30 μm.

**Supplementary Fig. 3**


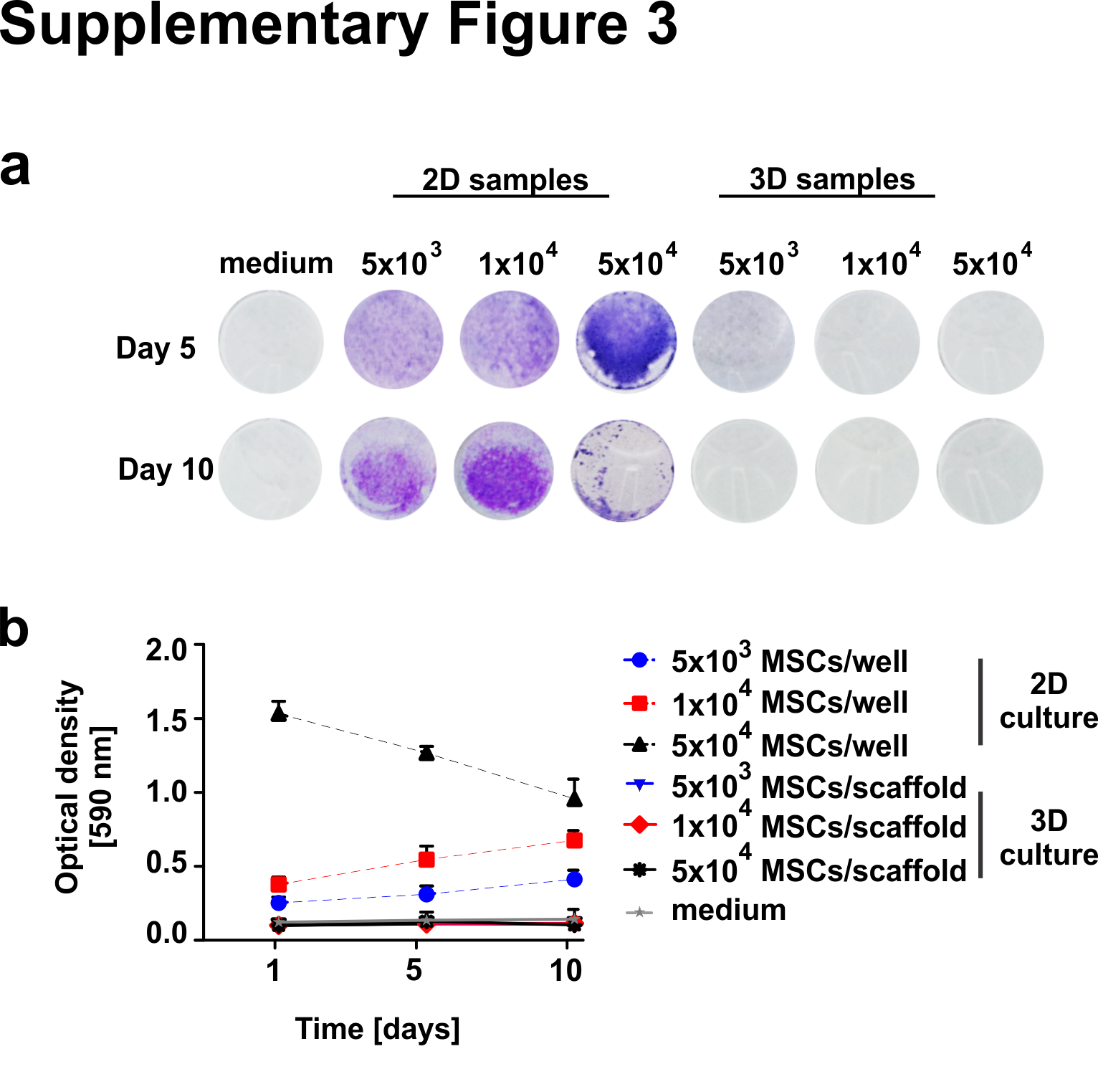


**Supplementary Fig. 3.** Adhesion of modified MSCs within the starPEG-heparin cryogel scaffolds. (a) Representative images of cell colony formation detected via crystal violet staining. Gene-modified MSCs were seeded at equivalent cell numbers in parallel in 2D or in cryogel scaffolds (3D) in the cavities of 96-well plates. At reported cultivation time points the cryogel scaffolds were removed and the cavities were stained with crystal violet to detect the growth of cells adherent to the walls. (b) Crystal violet dye of each well was dissolved in methanol and the optical density of the solutions was measured at 590 nm. The values attained from the wells housing the cryogel (3D) samples were compared to the ones of 2D samples and negative control (medium) to verify potential cell loss from scaffold over prolonged culture time. Data show the means ± SD of three independent experiments.

**Supplementary Fig. 4**

**
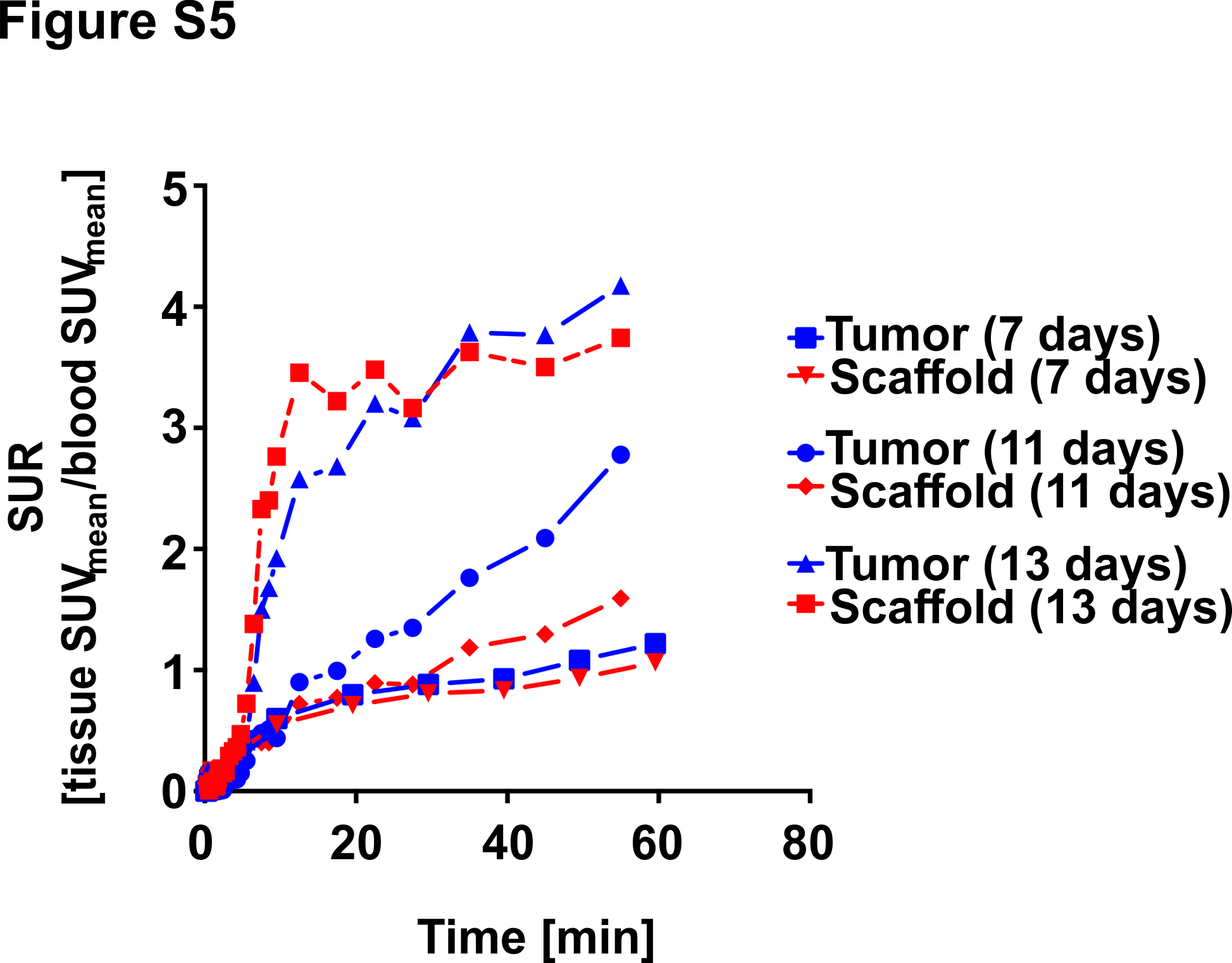
**

**Supplementary Fig. 4.** [^18^F]FDG-time-activity curves of the positron emission tomography (PET) study. The activity concentration is shown as standardized uptake ratio (SUR) for the cryogel scaffold and the tumor at day 7, 11, and 13 for one representative NMRI^nu/nu^ mouse out of 22.

**Supplementary Fig. 5**

**
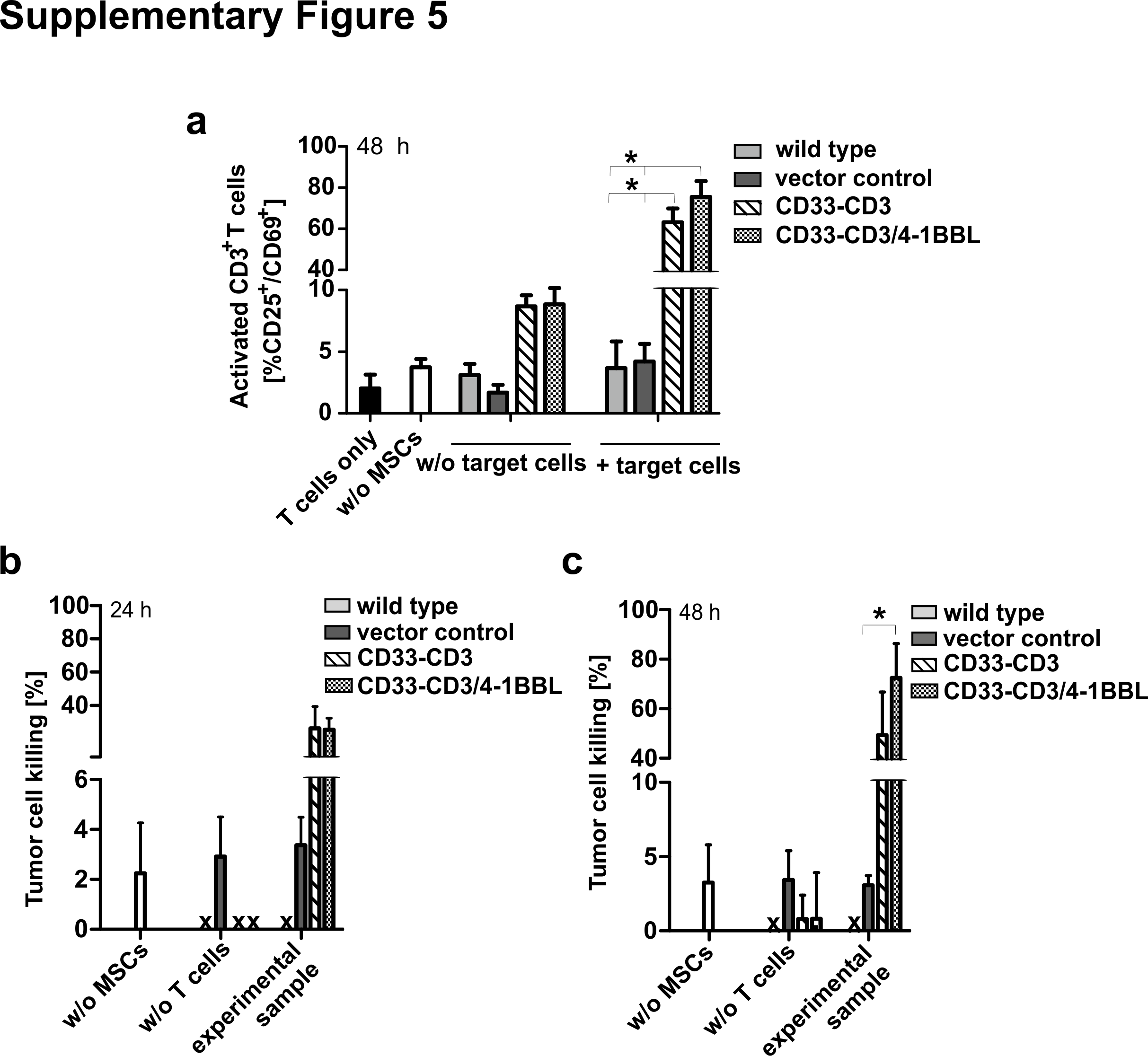
**

**Supplementary Fig. 5.** *In vitro* analysis of T cell activation and tumor cell killing via bsAb released by MSCs seeded in 2D as control. (a) T cells were incubated with 1x10^4^ CD33^+^ MOLM-13 cells at an effector-to-target (E:T) cell ratio of 1:1 in the presence or absence of 1x10^4^ 2D-seeded modified MSCs. Following 48h of co-cultivation, the percentage of CD25^+^/CD69^+^ cells on the total of CD3^+^ T cell number was determined via flow cytometry to evaluate specific T cell activation levels. (b-c) Specific lysis of ^51^Cr labeled CD33^+^ target cells detected after 24 h or 48 h of *in vitro* cultivation of 1x10^4^ T cells at an E:T ratio of 1:1 and 1x10^4^ bsAb-releasing or 4-1BBL expressing MSCs seeded in 2D. Data show the means ± SD of three different T cell donors. X= not detectable. **p*<0.05, one-way ANOVA/Bonferroni multiple comparison test.
